# Resilience Ontologies in Veterinary Science: How They Shape the Way We Address Resilience

**DOI:** 10.3390/vetsci13050471

**Published:** 2026-05-13

**Authors:** Hannah Keens Caballero, Heather Browning, Sarah Lambton, Damian Maye, Emma Roe

**Affiliations:** 1 School of Geography and Environmental Science, University of Southampton, Southampton SO17 1BJ, UK; h.keens-caballero@soton.ac.uk; 2 Department of Philosophy, University of Southampton, Southampton SO17 1BJ, UK; drheatherbrowning@gmail.com; 3 Bristol Veterinary School, University of Bristol, Langford BS40 5DU, UK; sarah.lambton@bristol.ac.uk; 4 Countryside and Community Research Institute, University of Gloucestershire, Cheltenham GL50 2RH, UK; dmaye@glos.ac.uk

**Keywords:** animal welfare, professional wellbeing, One Health, food system resilience

## Abstract

This narrative conceptual review aims to examine how veterinary science intertwines with the different ontologies of resilience. As resilience has increasingly become an influential yet conceptually diverse framework, its different ontologies shape and are shaped by veterinary science thinking. This paper will begin with a brief overview of the origins of the resilience concept and its three major ontologies: engineering, psychological, and ecological resilience. Following these different ontologies, the paper then explores animal-level resilience, where engineering framings emphasise disease response and production stability, while welfare-oriented perspectives frame resilience in terms of the affective experience and the lived realities of animals. It then considers veterinary professional resilience, highlighting how emotional labour, workload pressures and structural constraints shape wellbeing across the profession. Finally, it analyses how veterinary science contributes to socio-ecological resilience through One Health approaches in public health, food systems and climate adaptation. Across these domains, resilience is often framed as a desirable attribute, yet it remains a value-laden concept that can obscure inequities or normalise preventable harms. This paper calls for critical, justice-oriented engagement with resilience to ensure it supports ethically grounded veterinary practice and promotes healthier, happier animals, more equitable systems, and sustainable professional environments.

## Simple Summary

This narrative conceptual review explores how the concept of resilience shapes and is shaped by veterinary science in diverse ways. Resilience can refer to animals coping with disease, veterinary professionals managing stress, or whole systems like food production and public health adapting to challenges. By examining three main types of resilience—engineering, psychological, and ecological—the paper highlights how each shapes veterinary thinking and practice. It also argues that resilience is often treated as automatically positive, even though it can hide inequalities or place unfair burdens on individuals and therefore calls for a more critical approach to resilience in veterinary science.

## 1. Introduction

Veterinary science has had a long and complex history. As human–animal–environment relations have shifted from small scale farms to industrialised food production, from animals as workers to being members of the family, and through the effects of a changing climate and diet, veterinary science has adapted and continues to adapt to evolving landscapes of care and practice [1].

**Figure 1:**
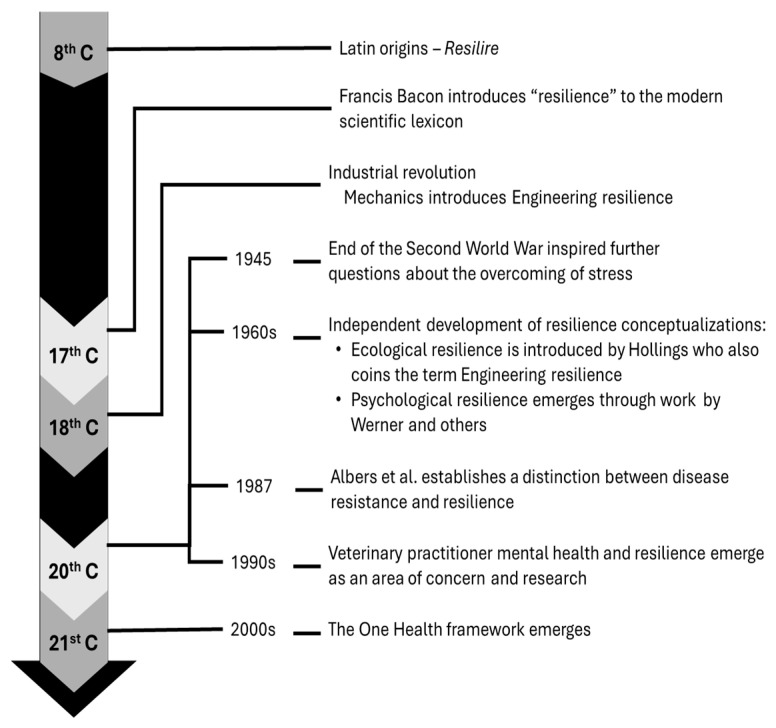
Timeline showing the historical development of resilience from its early origins to its expansion across engineering, ecological, and psychological disciplines and its adoption into veterinary science thinking.

**Figure 2:**
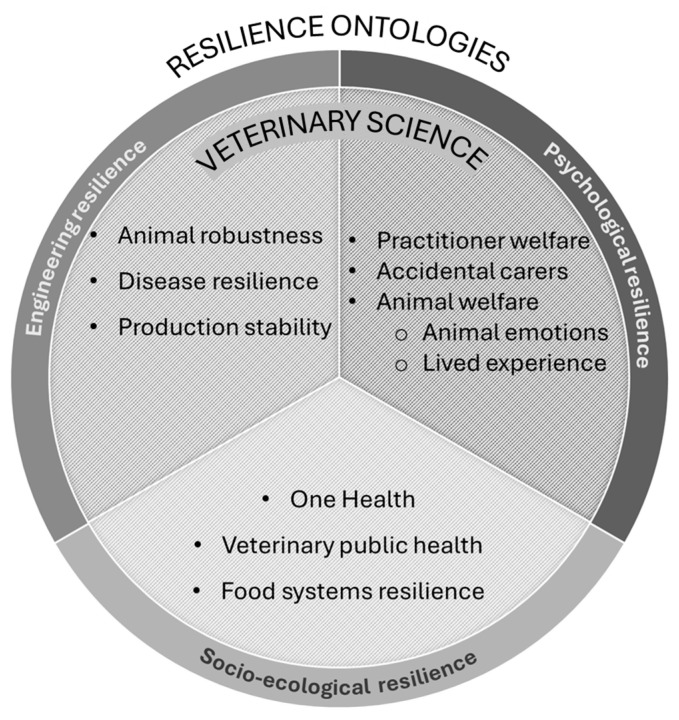
Conceptual map illustrating how veterinary science intersects with three major ontologies of resilience. Engineering resilience emphasises animal robustness, disease resilience and production stability; psychological resilience focuses on practitioner welfare, emotional labour, and animals’ lived experiences; and socio-ecological resilience encompasses One Health, veterinary public health and food systems resilience.

The concept of resilience has equally evolved over time (Figure 1) and has been broadened across disciplines [2], giving rise to multiple conceptualisations, known as ontologies, that although related, carry distinct implications for veterinary science thought and practice (Figure 2). These resilience ontologies must then be understood within their historical context, as they were shaped by the disciplinary priorities and societal demands of their time; and although veterinary science stands apart from these contexts, these ontologies continue to shape how resilience is interpreted and applied within the field.

Originally meaning “to jump back”, resilience was first used as a concept in modern science by Francis Bacon [2,3]. As the term has evolved, its literal understanding was left behind but its conceptual association with rebounding, recovery and redundancy can still be recognised in its different ontologies [4,5].

Engineering resilience is the first of the modern resilience ontologies; it emerged during the Industrial Revolution through the field of mechanics. Driven by a need to understand the properties of materials and machines, resilience described the elasticity and strength of materials under stress [2], focusing on how much stress an object or system could absorb before it failed and how readily it recovered. Importantly this conceptualisation holds the underlying assumption that there is a single “optimal” state towards which recovery should proceed [6] (Table 1).

During the early twentieth century, particularly in the context of the Second World War, resilience gained prominence and permeated from the fields of engineering and physics to other disciplines. In ecology, Hollings [7] reframed resilience to better fit the changing nature of ecosystems; unlike engineering resilience where there is a single equilibrium, ecological resilience refers to the capacity of a system to absorb disturbances while retaining core relationships, even when conditions shift. It acknowledges multiple stable states and emphasises thresholds, persistence, and transformation (Table 1). This framework later migrated into the social sciences, influencing sociology, economics, and geography [2] where it was then framed as socio-ecological resilience, expanding the same core assumptions from eco-systems to social systems [8] (Figure 2).

Psychological resilience [9] later emerged in the aftermath of the Second World War, as psychologists, influenced by earlier ontologies, began to consider whether the capacity of materials and ecosystems to absorb disturbance or return to an “optimal” state might also apply to the human mind. It subsequently became associated with an individual’s (particularly children’s) ability to adapt positively in the face of adversity [10,11] (Table 1).

Having examined the development and implications of different resilience ontologies it is then important to understand how they shape and are shaped by veterinary science. This narrative–conceptual review aims not to provide a comprehensive mapping of all the resilience literature in veterinary science, but to analyse how the different ontologies shape the understanding and application of resilience at animal, professional, and system levels. It further aims to critically evaluate the limitations of these framings and their influence on veterinary practice and policy.

## 2. Methodology

This study adopts a narrative–conceptual review approach developed through both exploratory and targeted literature engagement. Initial exploratory engagement with interdisciplinary resilience scholarship revealed that a wide range of disciplines contain substantial work dedicated to examining the origins, contexts, and conceptual implications of resilience. By contrast, although the term resilience is widely used within veterinary science, there appeared to be limited critical reflection on its underlying assumptions and meanings within the field.

To explore this further, a literature search was conducted using the Web of Science database, focusing on its Core Collection, MEDLINE, BIOSIS, and Zoological Record, which represent highly curated sources of veterinary research. The search term “resilience” was applied and refined to include only publications within the research area of “Veterinary Science” and those published in English. This initial search returned a large body of literature (2530 records), indicating the widespread use of the concept within veterinary research. A preliminary review of titles and abstracts suggested that, while resilience is frequently employed, its usage reflects multiple underlying conceptualisations consistent with the resilience ontologies identified in other disciplines.

Rather than conducting a systematic review, this study adopts an interpretive approach aimed at examining the conceptual implications of these differing ontologies. Targeted literature from adjacent disciplines was used to clarify the development and assumptions of the resilience ontologies. This was complemented by the non-systematic selection of veterinary literature addressing resilience at animal, professional, and system levels, with a focus on studies in which the concept is central to the analysis. To situate these conceptualisations within contemporary practice, additional grey literature from recognised veterinary institutions and adjacent disciplines was incorporated. This approach enabled a critical examination of how resilience ontologies are mobilised across veterinary research, practice, and policy.

## 3. Engineering Resilience

In veterinary science and animal production, one of the most established ways of understanding resilience comes from the engineering ontology of resilience (Figure 2). This framing was introduced to veterinary science through work on parasitic infection in sheep in the 1980s. Albers et al. [12] defined resilience as the ability to maintain a relatively undepressed production level when infected, explicitly differentiating it from resistance, the immunological capacity of resisting infection. As in engineering resilience, this new framing positioned productivity as that “optimal” state of an animal, with a direct or indirect relation to health.

Since then, engineering-based framings have continued to shape contemporary veterinary approaches to disease resilience and climate resilience in animals, especially through breeding and genetic manipulation. Here, resilience is understood as sustaining productive function under a wide range of multifactorial challenges [4,13], making resilience highly compatible with livestock production systems where performance under variability is highly valued.

This approach of resilience has a clear practical appeal. It makes resilience an operational process where deviations in production traits such as growth rate, weight gain or milk yield could be used as measurable indicators of an animal’s health and capacity to withstand disturbance [14]. These production metrics provide quantifiable proxies that align with industry priorities and support breeding and management strategies aimed at reducing antimicrobial use and improving robustness and efficiency in variable environments [15]. Recent advances in precision livestock farming have further embedded engineering resilience in the monitoring of animal performance. Continuous sensor-based data enable the detection of short-term deviations from expected production trajectories [14,16]. In poultry, for instance, daily fluctuations in body weight have been proposed as indicators of resilience, based on the assumption that more resilient individuals show smaller or more rapidly corrected deviations from predicted growth curves [17].

Despite its usefulness, this framing also carries conceptual risks. Within veterinary contexts, engineering resilience’s assumption of a single “optimal” state, against which deviations are measured and corrected, often translates into a productive baseline, where resilience is equated with the ability to maintain or return to expected levels of output. As a result, adaptive changes in behaviour or physiology may be interpreted as system failures rather than as legitimate coping strategies [14,16,18]. This creates a tension between productivity as resilience and adaptation as resilience.

This tension is particularly significant in relation to animal welfare. Stable or high production performance is not inherently indicative of good physical or psychological health, and animals may maintain performance while experiencing compromised welfare [19,20]. By seeking the “optimal” state, engineering approaches risk conflating productivity with health, and in turn welfare, narrowing the interpretation of resilience to what is measurable rather than what is meaningful from the animal’s perspective. Animal welfare science therefore pushes for a second lens through which to view resilience, drawing from a psychological resilience ontology. Here, rather than focusing on stability of output, this perspective of resilience encompasses emotional and motivational processes as well as physical performance, posing resilience as an animal’s capacity to maintain physiological homeostasis and engage in behavioural and affective processes associated with good welfare [14,20]. As Colditz [14] argues, resilience may be better understood as a competence to thrive, shifting the focus from recovery and control towards lived experience and quality of life.

Each of these framings emphasises different indicators, different welfare implications and different assumptions about what resilience looks like. Engineering resilience frameworks will remain a highly influential ontology within veterinary science particularly in production contexts. Recognising the limitations and assumptions of this ontology therefore remains critical as it directly shapes how resilience is measured, managed, and valued within veterinary practice and the livestock industry.

## 4. Psychological Resilience

The influence of the psychological ontology of resilience is increasingly evident within veterinary science. In contrast to engineering resilience, which prioritises stability and recovery to a single state, psychological resilience focuses on the emotional, cognitive and relational processes that enable individuals to cope with adversity (Table 1). This framing not only considers the capacity to withstand disturbance but also assigns positive value to the quality and direction of adaptive responses.

As discussed in the previous section, there has been a growing shift within veterinary science towards incorporating these psychological perspectives, particularly in relation to animal welfare, where increasing attention is given to lived experience and the pursuit of “positive” or “good” welfare and health [20] (Figure 2). While veterinary science frequently applies the concept of resilience to animals, the term is also widely used to address the people who work within and around the profession (Figure 2).

Drawing on psychological conceptualisations, this body of scholarship has become increasingly important as evidence accumulates of elevated rates of burnout, anxiety, depression, compassion fatigue and suicide among veterinary professionals, placing practitioner wellbeing as a central concern in the contemporary professional discourse [21,22].

Across both clinical and agricultural settings, veterinarians routinely perform roles that extend well beyond the provision of medical care. They often act as educators, advisors, communicators, and sources of emotional support. These additional responsibilities are integral to how veterinary work is delivered, yet they contribute to the psychosocial pressures faced by its professionals [23]. In contexts of animal production and care, particularly in rural settings, formal mental health services are often limited or stigmatised, leading veterinarians to frequently become “accidental counsellors”, as farmers confide distress in those they see regularly and trust [24].

In veterinary clinical practice, this demand is further intensified. Veterinarians perform emotional labour when helping owners navigate guilt, grief and moral conflict in the context of medical decision making and euthanasia [25]. Additionally, veterinarians also occupy a sentinel role in identifying animal abuse and associated human interpersonal violence [26,27]. These forms of informal emotional and safeguarding work constitute meaningful, though often unrecognised, ways in which veterinary professionals shape resilience at the level of households and communities.

The emotional demands of veterinary work, combined with other pressures, intrinsic to the profession, such as client expectations, financial insecurity, student debt, workload intensity, and concerns about ecological change, place significant strain on practitioners’ resilience [21,28]. These pressures also prominently affect veterinary students and early career veterinarians, who may have limited experience in managing the ethical and emotional challenges of the profession [29,30]. In response, regulatory bodies have increasingly framed wellbeing and resilience as components of professional competence. Initiatives such as the Royal College of Veterinary Surgeons’ Mind Matters Initiative [31], the Australian Veterinary Board Council’s Day One Competences [32], and the American Veterinary Medical Association’s Annual Health and Wellness Summit [33], aim to provide training and support to enhance mental health and resilience across the veterinary profession.

Despite this growing attention, much of the literature focuses on the factors that erode resilience rather than those that enable it. Cake et al. [21] highlight that relatively little work examines the positive psychological, social and organisational factors that enable resilience to be developed and sustained. This is echoed by One Welfare scholarship, which argues that while veterinarians are expected to contribute to broader safeguarding systems, many lack appropriate training and institutional support, placing responsibility on the individuals rather than on the institutions responsible for supporting them [27]. Studies consistently show that supportive team dynamics, mentorship, opportunities for rest and recovery, and manageable workloads are critical protective elements, emphasising the relational and contextual nature of resilience within veterinary practice [29,34].

These gaps reflect a broader limitation of psychological framings of resilience when applied uncritically within veterinary contexts. When resilience is treated primarily as an individual responsibility, structural constraints and systemic pressures may be obscured. As a result, the burden of adaptation risks being placed disproportionately on practitioners and, in the context of animal welfare, on individual animals. While psychological approaches offer an important lens for framing resilience, their uncritical application risks reinforcing existing inequities. Recognising these limitations underscores the need for systemic and organisational interventions, rather than relying solely on individual coping strategies.

## 5. Socio-Ecological Resilience

The rise in integrative and multidisciplinary approaches that highlight the interdependence of human, animal, and environmental health positions veterinary science as a critical actor within a broader resilience-building process. In contrast with engineering and psychological resilience which focus on stability or individual coping, these integrative approaches frame resilience through a (socio-)ecological ontology where addressing interactions and relationships leads to adaptation across multiple scales (Table 1).

Veterinary science has been central to the rise in these integrative approaches, most notably through the development of the One Health perspective (Figure 2). The origins of the One Health framework lie in veterinarian science, stemming from Calvin Schwabe’s [35] and other veterinarians’ call for the unity of human and veterinary medicine (One Medicine). This approach was later expanded through socio-ecological frameworks [36] into One Health systemic thinking. While resilience is not always explicitly invoked within the One Health literature, it is increasingly embedded in its logic and application.

Indeed, international organisations such as the World Organisation for Animal Health (WOAH) [37] and the Lancet’s One Health Commission [38] frame core veterinary activities, such as surveillance, biosecurity, antimicrobial stewardship, and zoonotic disease control, as central to strengthening the resilience of health systems [37,38,39]. Nonetheless, as Nitzan et al. [40] argue, there might be a need for more explicit incorporation of resilience into One Health governance.

Within the socio-ecological framing, veterinary science contributes to resilience across multiple interconnected domains. Veterinary public health interventions such as rabies control programmes, combining mass dog vaccination and integrated surveillance across human and animal health systems, have reduced human disease burden while strengthening health system stability [41]. Similarly, climate-driven shifts in wildlife disease patterns further emphasise the need for ecological surveillance and veterinary engagement in preventative measures, underscoring the role of veterinary science in mediating the impacts of environmental change on both animal and human health systems [42].

Veterinary science also plays a critical role in the resilience of food systems. For example, regulatory interventions such as Denmark’s Yellow Card scheme have produced sustained reductions in antimicrobial use in pig herds [43]. In addition, work on climate mitigation shows that improving animal health can reduce greenhouse gas emissions per unit of product, highlighting resilience pathways that benefit both environmental and production outcomes [44]. Further research on sustainable production methods distinctly influences modern intensification, species choice and management practices [45].

Empirical research from crisis settings further illustrates this role. During periods of disruption, such as armed conflict or natural disasters, veterinary services contribute to maintaining food production, supporting livelihoods, and enabling adaptive responses at both farm and community levels. Studies from Ukraine, for example, demonstrate how decentralised expertise and adaptive management practices allowed farmers and veterinarians to sustain production under conditions of infrastructural instability [46]. At the household level, veterinarians also contribute to resilience through routine interactions with animal owners, with research indicating that they are trusted sources of information on disaster preparedness, thereby strengthening community-level resilience capacities [47].

However, while veterinary science contributes to resilience across these domains, its own position within socio-ecological systems reveals important limitations and tensions. Participation in global resilience efforts remains uneven, with disparities in infrastructure, funding, and institutional capacity limiting the ability of veterinary services to engage fully in international crisis management [37]. At the same time, the environmental footprint of veterinary practice itself remains underexamined, raising questions about the sustainability of the systems it supports [48].

While veterinary science is positioned as a central actor in resilience building, the extent to which it can fulfil this role depends on broader institutional, economic, and environmental conditions. These dynamics highlight a key feature of socio-ecological resilience, as it distributes responsibility across systems and relations; however, it does so while potentially obscuring inequalities in capacity and burden. Recognising this is essential, as it shifts resilience from a descriptive concept to a normative framework that shapes how responsibilities, resources, and risks are allocated within veterinary science and the systems in which it operates.

## 6. Discussion

This narrative–conceptual review has explored the many ways in which resilience intertwines with veterinary science across multiple domains (Figure 2). As outlined in Table 1 each ontology answers different questions about what should persist, who should adapt, and which outcomes should be valued [49,50,51]. Consequently, questions such as who determines what should be resilient, how resilience is achieved, what constitutes a desirable outcome, and who bears the costs of adaptation are central to understanding the consequences of resilience framings [5,9,51,52,53,54].

Within both veterinary science and the wider literature, resilience is frequently presented as an inherently positive property [14,52,55]. However, resilience does not necessarily equate to desirability. A classic ecological example is the persistence of a turbid lake, which, despite being an unfavourable state for an ecosystem, can remain highly resilient [49]. Similar dynamics can be observed in livestock production, where animals bred for resilience may maintain performance under stress while still experiencing compromised welfare [4]. These examples illustrate that resilience can stabilise undesirable states rather than enable their transformation.

The analysis presented in this review shows that veterinary applications of resilience reflect broader critiques identified across wider resilience scholarship. Scholars across disciplines have called for the explicit incorporation of justice and equity into conceptualisations of resilience. In climate resilience research, the equitable resilience framework [54] emerged to address the persistent inequalities produced by policy approaches that burden already vulnerable and marginalised communities with disproportionate adaptation responsibilities, with parallel critiques appearing in disaster management studies [56,57]. These concerns reflect the limitations associated with socio-ecological framings of resilience identified in this review, particularly in relation to the distribution of responsibility and capacity.

Similarly, the limitations of psychological framings highlighted in this review echo concerns raised in other disciplines. In health services, resilience discourse has been shown to reinforce normative assumptions about whose suffering counts and whose adaptation is expected [53], demonstrating how resilience framings may penalise individuals whose responses to adversity deviate from dominant expectations [9]. In this sense, both psychological and socio-ecological ontologies carry implicit assumptions that can shape how responsibility for adaptation is allocated.

This critical stance is equally important for veterinary science. While veterinary scholarship has significantly engaged with measuring resilience in animals, professionals, and systems [14,16,19,58], this review finds comparatively limited critical attention given to the normative assumptions embedded in these approaches. Veterinary professionals act as advocates for animal health and welfare, placing them in a key position to interrogate normative framings of resilience in animals. At the same time, the profession operates within shifting social and structural contexts, including its ongoing feminisation [59]; these challenges require a clearer understanding of what is normalised within the resilience discourse.

Veterinary science also regularly intersects with marginalised communities whose adaptive capacities are constrained. These communities often rely heavily on veterinary services while facing structural barriers that shape their ability to respond to challenges [60]. In such contexts, resilience framings may inadvertently shift responsibility onto individuals, whether practitioners, animal owners, or animals themselves, without addressing underlying systemic constraints. Recognising the tensions that emerge from different ontologies is therefore essential if veterinary science is to engage critically and ethically with resilience as both a scientific and normative concept.

## 7. Conclusions

This review has demonstrated that resilience is not a singular or neutral concept within veterinary science, but a framework shaped by distinct ontologies that carry different assumptions, priorities, and implications. By tracing the development of engineering, psychological, and socio-ecological resilience, this paper shows how each ontology structures the way resilience is understood and applied across animal health, veterinary professional wellbeing, and broader socio-ecological systems.

In animals, the dominance of engineering framings has aligned resilience with productivity and stability, often at the expense of welfare considerations. In contrast, psychological approaches have introduced a focus on lived experience and adaptive capacity, but risk shifting responsibility onto individuals, whether animals or practitioners, while obscuring structural determinants. At the systems level, socio-ecological resilience highlights the interconnected role of veterinary science in global health and food systems, yet similarly raises questions around equity, responsibility, and uneven capacity.

Taken together, these findings show that resilience in veterinary science operates not only as an analytical concept, but as a normative framework that shapes what is valued, measured, and prioritised within veterinary science. Its application can illuminate vulnerability and support adaptation, but it can also obscure structural inequalities and normalise undesirable conditions if applied uncritically.

This paper therefore argues that veterinary science must engage more explicitly with the assumptions underlying resilience framings. Rather than adopting resilience as an inherently positive goal, a critical and reflexive approach is required—one that recognises whose resilience is being prioritised, under what conditions, and at what cost. Such an approach is essential if resilience is to support ethically grounded veterinary practice, promote meaningful animal welfare, and contribute to more equitable and sustainable systems.

## Figures and Tables

**Table 1:** Summary of the main resilience ontologies, outlining their historical origins, key assumptions, conceptual characteristics, their implications and their risks for veterinary science.

*Resilience Ontology*	*Core Assumptions*	*Key Characteristics*	*Veterinary Relevance*	*Limitations/Risks*
*Engineering* *Resilience*	Objects, materials and/or systems have a single optimal state to which they should return after disturbance	Stability, resistance, recovery, elasticity, robustness	Disease resilience, production stability, livestock performance, breeding for robustness	May conflate performance measures as single indicators of health and welfare
*(Socio-)Ecological* *Resilience*	Systems can exist in multiple stable states and adapt to change	Adaptation, transformation, thresholds, persistence, system dynamics	One Health, food systems resilience, ecosystem health, climate adaptation	May obscure systemic inequalities and normalise undesirable states
*Psychological* *Resilience*	Individuals can mentally adapt positively to adversity and maintain wellbeing	Coping, adaptation, emotional regulation, recovery, thriving	Veterinary professional wellbeing, burnout, emotional labour; animal welfare and affect	May place undue burden on individuals to cope with broader systemic conditions

## Data Availability

No new data were created or analyzed in this study. Data sharing is not applicable to this article.
